# Developing and evaluating training for pharmacists to offer a pharmacy-led public health service in primary care

**DOI:** 10.1186/s40545-022-00480-6

**Published:** 2022-11-18

**Authors:** Aliki Peletidi, Reem Kayyali

**Affiliations:** 1 Pharmacy Programme, Department of Health Sciences, School of Life and Health Sciences, University of Nicosia, Nicosia, Cyprus; 2 Department of Pharmacy, School of Life Sciences, Pharmacy and Chemistry, Kingston University, London, UK; 3 School of Pharmacy, Newcastle University, Newcastle Upon Tyne, UK

**Keywords:** Pharmacy-led services, Pharmacists’ training model, Greece, Weight management

## Abstract

**Background:**

Currently in Greece, no formal organisation offers post-academic education to pharmacists. To improve the clinical practice of pharmacists, there is a need for training that will educate them on how to approach and consult their patients. The study aimed to evaluate the training required by pharmacists to offer a pharmacy-led weight management service in Greece.

**Methods:**

The study consisted of 3 phases. During the first phase educational needs of the participating pharmacists were identified. A pre-training quiz was given consisting of 14 questions to investigate the participating pharmacists’ knowledge on guidance, causes and facts of obesity. The second phase comprised the training design and delivery. The final phase dealt with training evaluation (27 questions in total), which included the perceived rating of knowledge and confidence levels pre- and post-training, and a post-training quiz (the same as the pre-training quiz). The post-evaluation questionnaire and the quiz (pre- and post-training) results were entered into SPSS Version 23 for statistical analysis.

**Results:**

The mean total quiz score was 6.38 (SD = 2.56) pre-training compared to 11.92 (SD = 1.20) post-training (*P* < 0.001). Nearly all community pharmacists, 96.2% (*n* = 25) stated that they had an excellent/good experience and 88.5% (*n* = 23) strongly agreed/agreed that their expectations were met.

**Conclusions:**

Training delivered was well received and it enhanced participants’ knowledge on the topic. Limitations include the small number of participating pharmacists and investigation of short-term training effects only. Due to the positive outcomes of the training, however, it has the potential to become a model for Greek pharmacists to offer different pharmacy-led public health services.

## Background

Greece is in South-Eastern Europe. According to the latest census conducted in 2021, the total population of Greece was estimated at 10,432,481–5,075,249 males and 5,357,232 females [1]. In addition, the latest estimated life expectancy in Greece (in 2020) is 65 years for men and 66.8 years for women [2].

The Organisation for Economic Co-operation and Development (OECD) [3] stated that self-reported obesity among adults in Greece is at 17%. In addition, data from the first National Survey of Morbidity and Risk Factors (*EMENO*) project [4] reports that the percentages of overweight and obese people overall among the Greek population are at 36% (43% in men and 30% in women) and 32% (30% in men and 34% in women), respectively. The study revealed that Achaia, which is the capital of Peloponnese [5], held the highest percentages in overweight (38% overall, 42% in men and 32% in women) and obese (36% overall, 35% in men and 38% in women) citizens compared with the rest of Greece.

Behavioural change is important in improving healthcare and health outcomes [6]. The Jackson et al. study [7] emphasised that healthcare professionals (HCPs) are in a great position to play a key role in helping people make a behavioural change in relation to weight management (WM). Furthermore, a study by Rose et al. [8] added that HCPs in primary care could give effective advice on weight loss, and that this could lead to behavioural change thus leading to positive weight reduction. However, a big obstacle to improving public health is the inability of HCPs to apply their knowledge appropriately [9, 10], something that echoes the Peletidi et al. study [11], which aimed at exploring the views of the pharmacy-led WM service providers in England. Therefore, training to improve knowledge including communication skills is essential [10, 12–16].

According to previous published data [17], Greek pharmacists tend to use a paternalistic approach in their consultations, which does not favour the patient-centred approach. Generally, pharmacists lack knowledge and confidence, both in communication and motivational skills [7, 18], as well as in approaching people and helping them make a change. Currently in Greece, no formal organisation offers post-academic education to pharmacists. Kostagiolas et al. [19] expressed the need for a library of information services to be created so that Greek pharmacists could improve their knowledge and skills. Greek pharmacists who indicated that they act mainly based on their experiences [17]. A lack of knowledge, skills, and resources, do not allow Greek pharmacists to approach and counsel their patients in the way that they should. To improve the clinical practice of Greek pharmacists, there is a need for training that will educate them on how to approach and consult their patients. Furthermore, according to Walters et al. [20], training pharmacists can influence the initiation and maintenance of providing services. McCormick et al. [21] indicated that such training displays a “protective” role by minimising participants’ withdrawal from services.

In general, there are two predominant learning methods: passive learning (teacher-centred paradigm) and active learning (learning-centred paradigm) [22]. The Anderson et al. study [10] reported that employing traditional training methods (passive learning) in the pharmacy profession is unsuccessful, as it is not thoroughly related to practice competencies in accordance to people’s needs. According to the Kocla-Kimble et al. study [23], which designed an active learning model for pharmacists offering diabetes care, involving role-play as an activity during their training, pharmacists showed an increase in their problem-solving abilities, their communication skills and their self-confidence. In addition, group discussion proved to be a great way of exchanging ideas amongst learners.

Therefore, this study aimed to identify and explore the current knowledge and educational needs and preferences of Greek pharmacists as well as to design, deliver and evaluate the training required by Greek pharmacists to offer a WM programme through their pharmacies.

## Methods

Based on the findings of Peletidi et al. study [17], the decision to deliver a WM programme in Greece was taken and more specifically in Patras (the third largest city in Greece). Greece was primarily chosen based on convenience due to local knowledge, contacts and ease of collaboration. The population of the municipality of Patras in 2011 was estimated at 213,984 (104,307 males and 109,677 females) [24].

In Western Greece, 624 pharmacies account for 6.01% of the total number of pharmacies. Specifically, according to the Pharmaceutical Association of Achaia, 223 pharmacies are located within the municipality of Patras. The Pharmaceutical Association of Achaia informed pharmacists in Patras (through an e-mail sent by them) about the service, to determine their interest in participating in it. Training was essential for the service delivery. The following phases were completed as part of the training provision; Phase I investigated the educational needs of pharmacists, their knowledge levels, and how they approach overweight and obese people. Phase II comprised the design and delivery of the training and Phase III evaluated the training delivered.

Before all stages of the study, all relevant documentation (Appendix 3) were ethically approved by the Ethics Committee of Kingston University (Faculty of Health, Science, Social Care and Education) London (Ref: 1516/030), as well as by the Pharmaceutical Association of Achaia (Ref No. 56, 23/3/2016). In addition, all materials were validated and piloted by three Greek pharmacists with no additional changes. The structure and materials developed for the WM programme are explained in Peletidi et al. study published earlier [25].

### Phase I: educational needs assessment

#### Participants’ selection

All pharmacists in Patras were informed, through the Pharmaceutical Association of Achaia, about the WM programme delivery via e-mail. The interested pharmacists who responded positively to this e-mail were provided with further communication. The pharmacist-researcher went to each of the interested pharmacies in person to give further details about the programme. In total, 26 pharmacists responded positively and agreed to participate in the WM programme.

#### Pre-training quiz

Participants were asked to complete a pre-training quiz. The quiz aimed to determine pharmacists’ level of awareness and knowledge on obesity and WM, as well as their readiness to work with and assess obese people. The quiz included 13 multiple-choice questions (note that Question 5 contained two sub-questions) related to obesity statistics, nutritional and physical activity guidelines, and motivational interviewing skills. The questions were based on the European Society of Cardiology (ESC) Clinical Practice Guidelines and the World Health Organization (WHO) guidelines on obesity [26, 27]. The estimated time that took pharmacists to complete the quiz was 10 min.

### Phase II: design and delivery of the training

The training was based on the findings identified in the interviews conducted with UK pharmacists and pharmacy staff who underwent training prior to offering a WM programme [11]. The content of the training sessions was based on the educational needs that were reported by participating pharmacists. The content was also based on the findings identified in the interview analysis of UK pharmacists and pharmacy staff who offered the WM service [11]. The training sessions were mainly designed based on active learning methods, which included role-playing, group activities and discussion. Lecturing was kept to a minimum.

The instructor of the training was a pharmacist who underwent specialised training in obesity management before delivering the training for the participating pharmacists. It was also important that the training was trainee-centred as it was essential for participating pharmacists to prepare themselves for offering the WM programme.

**Fig. 1:**
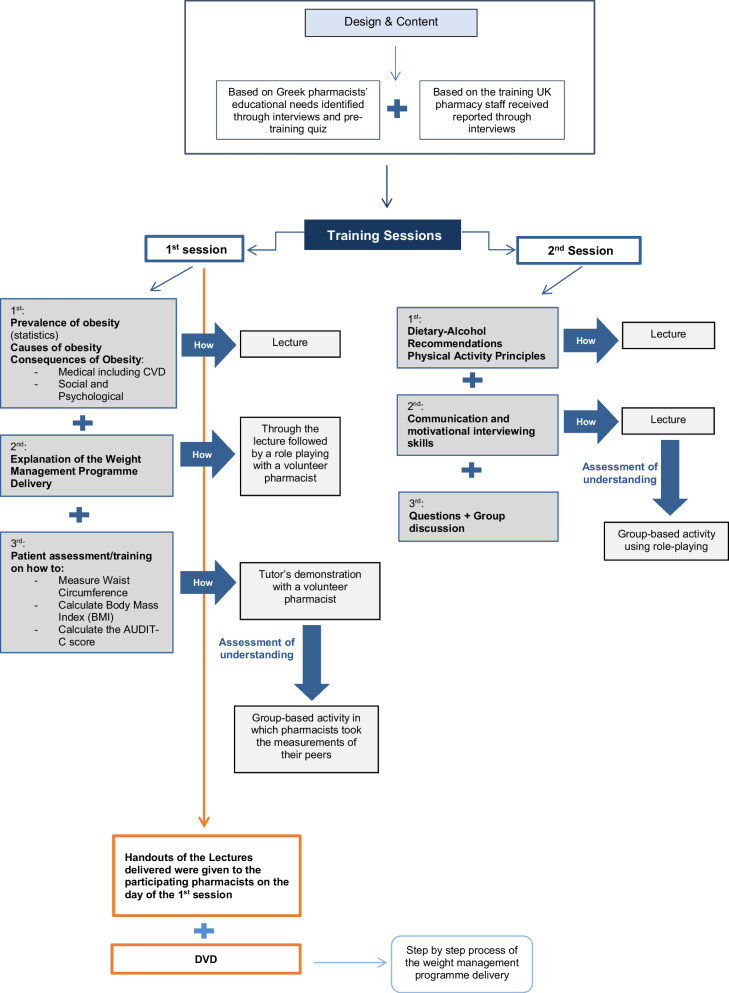
Diagrammatic explanation of the training sessions delivered

The training was split into two evening sessions and was delivered by the trained pharmacist-researcher. The training was conducted in person and took place at the headquarters of the Pharmaceutical Association of Achaia, located in Patras. Each session lasted 3 h and was carried out in the form of interactive lectures (Fig. 1). In addition, the training also included active learning techniques such as role-playing and group activities, followed by a group discussion.

The training was based on the guidelines on CVD prevention in clinical practice developed by the ESC [26]. Pharmacists were also trained on conducting patient assessments for the WM service, i.e. questions required such as age, gender, smoking history, exercise and diet habits, and measurements/calculations such as weight, height, waist circumference (WC), blood pressure (BP) and the alcohol use disorders identification test (AUDIT-C) score [28]. The patient assessment was based on assessments conducted in the UK in various public health services such as the National Health Service (NHS) health checks [29]. First, the tutor-researcher requested a participant to volunteer so that the measurement-taking method could be demonstrated to the rest of the participants. Afterwards, they practised by working in groups, and taking each other’s measurements.

Furthermore, a DVD was also given to the participating pharmacists. It was designed and recorded in the Greek language, depicting the process of the WM programme step-by-step. The pharmacist-leader (researcher) role-played a pharmacist, and volunteers role-played participants, in this way mimicking the service’s procedure. Finally, training handouts, including the lecture slides that were delivered in the sessions, and the DVD, were distributed to pharmacists on the day of the first training session, as requested during the pre-training interviews.

### Phase III: post-training quiz and post-training evaluation questionnaire

A post-training quiz (the same quiz used during pre-training) was distributed to attending pharmacists to evaluate what they learned throughout their training on obesity and WM. The quiz was completed in the training venue without them using the lecture handouts that were provided earlier.

A post-training evaluation questionnaire was also distributed so that pharmacists could provide overall feedback on the training they received and to determine their views on it. The questionnaire was developed based on previous studies that evaluated pharmacists’ training [12, 30, 31]. The questionnaire comprised 27 questions (included open-ended questions and 5-Point Likert-scale type questions) that were divided into 8 subsections: the pre-training quiz, the training sessions, the teaching materials, the learning approach, the tutor, the venue, and the facilities. The last section of the questionnaire included demographic questions related to age, gender, years of experience and pharmacists’ learner types based on the VARK model of learning styles (visual, auditory, reading/writing, and kinesthetic) [32, 33].

In the questionnaire, two questions regarding pharmacists’ knowledge and confidence level before and after the training, consisting of 12 and 7 statements, respectively, were included (Table 1). The average time recorded for completing the post-training evaluation questionnaire was estimated at 20 min, whereas the quiz was completed in 5–7 min.

**Table 1:** Statements included in the questionnaire, related to confidence and knowledge (Q5&7)

Questions			
Q5	Confidence level	Q7	Knowledge level
(a)	I have confidence in approaching people	(a)	Scale of obesity
(b)	I am confident to assess people’s weight	(b)	Consequences of obesity
(c)	I am confident to measure people’s waist circumference	(c)	Recommended daily calories intake
(d)	I am confident to measure blood pressure and heart rate	(d)	Daily intake of foods per food groups
(e)	I have confidence and I am able to motivate people	(e)	Dietary recommendations
(f)	I am confident to discuss and set personalised goals with them	(f)	Alcohol consumption limits
(g)	I am confident to follow them and to provide lifestyle advice	(g)	Calculation of BMI
		(h)	Measurement of waist circumference
		(i)	How to use AUDIT-C score
		(j)	Physical activity guidelines
		(k)	Motivational interviews and consultation skills
		(l)	Weight management programme overall

### Data analysis

All data for both the post-evaluation questionnaire and the quiz (pre- and post-training) were entered into IBM Statistical Package for Social Sciences (SPSS) Version 23 for statistical analysis. Descriptive statistics were presented as frequencies and percentages of participants’ responses. Data presented as descriptive statistics were exported into Microsoft Excel to produce graphs.

### Post-training evaluation questionnaire (knowledge and confidence level questions)

Participants’ responses relating to rating of their knowledge and confidence level were presented as frequencies and percentages of participants’ responses. The mean, the standard deviation (SD) and the median of the rating of each statement was also provided [34] both before and after the training.

In addition, to assess pharmacists’ knowledge and confidence levels before and after the training, inferential statistics were conducted. A priori level of statistical significance was set at a *P*-value less than 0.05. The reliability (internal consistency) of the Likert-scale questions regarding pharmacists’ knowledge and confidence level was assessed by calculating Cronbach’s Alpha coefficient. In general, a scale is deemed to have a good internal consistency when the Cronbach’s Alpha coefficient is greater than 0.7 [35, 36]. To examine the statistical significance difference in pharmacists’ knowledge and confidence level before and after the training for each statement, the sign test (non-parametric test) was conducted. Furthermore, to examine the overall effect of the training on pharmacists’ knowledge and confidence levels, the paired-samples *t*-test was used. The normality assumption regarding the difference in the total scores before and after the training was checked via the Shapiro–Wilk test [37] before using the *t*-test.

### Pre- and post-training quiz

Additionally, to examine the training’s effects on pharmacists’ response rates for each question included in the quiz, the McNemar’s test was utilised. Furthermore, to examine the effect of training on the overall pharmacists’ knowledge level, the paired sample *t*-test was used. The pharmacists’ total quiz scores before and after the training were calculated. The minimum total score that a pharmacist could achieve was 0 and the maximum was 14. The normality assumption regarding the difference of the total scores before and after the training was tested using the Shapiro–Wilk test [37].

### Effect of demographics on pharmacists’ knowledge, confidence and quiz scores

The effect of each of the demographic factors (gender, age, years of experience and type of learner) on pharmacists’ total scores for perceived knowledge and confidence levels as well as their quiz total scores before and after the training were measured. An unpaired samples *t*-test was conducted to assess the impact of gender (male, female) on knowledge level, confidence level, and quiz performance both before and after the training. The normality assumption was tested using the Shapiro–Wilk test [37]. A one-way ANOVA was conducted to assess the impact of age, years of experience and learner type (auditory, visual, reading and writing) on pharmacists’ knowledge level, confidence level and quiz performance both before and after the training.

## Results

Most participants were males, comprising 61.5% (*n* = 16) of the sample. Most participants were aged between 35 and 44 years (38.5%) (Table 2). Their experience varied between ≤ 5 years (26.9%) to more than 20 years (30.8%). All participants owned a pharmacy in Patras. Their learner types varied, with the majority being reading/writing learners (46.2%), as opposed to the minority being auditory learners (23.1%) (Table 2).

**Table 2:** Personal demographic information (*n* = 26)

	Frequency (*n*)	Percentage (%)
Gender distribution		
Sex		
Males	16	61.5
Females	10	38.5
Age distribution		
Age range		
25–34 years	7	26.9
35–44 years	10	38.5
45–54 years	5	19.2
55–69 years	4	15.4
Years of experience		
Year range		
≤ 5 years	7	26.9
6–10 years	5	19.2
11–15 years	4	15.4
16–20 years	2	7.7
> 20 years	8	30.8
Type of learner		
Visual learner	8	30.8
Auditory learner	6	23.1
Reading/writing learner	12	46.2

### Pre-training quiz

A pre-training quiz was circulated to assess the knowledge of the participating pharmacists on obesity and WM. Pharmacists were asked to answer 13 questions regarding obesity, WM, and motivational interviewing. The total number of pharmacists who responded correctly to each question is provided in Table 3 and the total quiz score of each pharmacist is provided in Table 4. The highest score was 11/13, achieved by 2 pharmacists, while the lowest score was 1/13, achieved by 1 pharmacist.

**Table 3:** Total number of participants who responded with either correct or incorrect in the pre-training quiz

Questions		Correct (*n*)	Wrong (*n*)
Question numbers	Question statements	Pre-training	
Q2	What causes obesity?	23	3
Q6	What is the acceptable daily intake of saturated fats?	19	7
Q8	An obese person has excess weight that includes muscle, bone fat and water	16	10
Q3	How much is the Body Mass Index (BMI) range that indicates that a person is obese?	13	13
Q12	How much is the recommended weekly physical activity for an average adult, according to the European Society of Cardiology (ESC) guidelines?	13	13
Q4	What is the recommended daily fruit and vegetable intake for an average adult according to the European Society of Cardiology (ESC) guidelines?	12	14
Q10	Alcohol contains more calories per unit weight than carbohydrates	12	14
Q11	How many calories does a medium glass of red wine (175 ml) have?	11	15
Q9	What is the alcohol daily limit for an adult male according to the European Society of Cardiology (ESC) guidelines?	10	16
Q1	How many adults in Greece were classified as obese according to World Health Organization (WHO)?	9	17
*Q5*	(*b*)* What is the recommended daily calorie intake for an average adult male?*	*8*	*18*
	(*a*)* What is the recommended daily calorie intake for an average adult female?*	*7*	*19*
*Q13*	*Motivational interviewing deals with which of the following four points?*	*7*	*19*
*Q7*	*Some fats have been shown to increase cardiovascular disease risk. Which one*(*s*)*?*	*6*	*20*

The questions are organised in terms of frequency of correct answers. The underline highlighting indicates the top three questions that the majority of pharmacists responded correctly to, while italics indicates the three lowest

**Table 4:** Pharmacists’ total score pre-training

Pharmacist code	Total score pre-training
WM 1	3
WM 2	4
WM 3	8
WM 4	5
WM 5	7
WM 6	7
WM 7	10
WM 8	11
WM 9	8
WM 10	4
WM 11	7
WM 12	7
*WM 13*	*1*
WM 14	7
WM 15	4
WM 16	7
WM 17	11
WM 18	8
WM 19	6
WM 20	3
WM 21	4
WM 22	8
WM 23	7
WM 24	9
WM 25	7
WM 26	3

Italics highlighting indicates the minimum total score observed and underline the maximum

### Phase III: post-training evaluation questionnaire and quiz

#### Overall pharmacists’ feedback of the training

Upon completion of the training, a post-training evaluation questionnaire was given to the participating pharmacists (*n* = 26) to provide overall feedback on the training they received. Results are described in Tables 5, 6 and 7. In terms of their overall training experience, 96.2% (*n* = 25) stated that they had an excellent/good experience (Table 5) while only 3.8% (*n* = 1) shared that their overall experience was average.

**Table 5:** The participants’ rating of various aspects of the training (N = 26)

Statements	Excellent	Good	Average	Below average	Poor
	1	2	3	4	5
	Frequency (*n*)/percentage (%)				
Training and training materials					
How would you rate your overall training experience	10 / 38.5	15/ 57.7	1/ 3.8	–	–
How would you rate the training materials provided prior to the training?	19/ 73.1	7/ 26.9	–	–	–
How would you rate the structure of the training materials delivered during the session?	18 /69.2	7/ 26.9	1/ 3.8	–	–
The visual aids and handouts provided	19/ 73.1	6/ 23.1	1/ 3.8	–	–
The video provided	16/ 61.5	7/ 26.9	2/ 7.7	1/ 3.8	–
The role-playing	13/ 50	8/ 30.8	4/ 15.4	1/ 3.8	
The case studies	11/ 42.3	13/ 50	2/ 7.7	–	–
The group discussion	10/ 38.5	12/ 46.2	3/ 11.5	1/ 3.8	–
Venue and equipment					
The venue used for the type of training provided was sufficient	11/ 42.3	13/ 50	2/ 7.7	–	–
The venue meets my expectations	12/ 46.2	12/ 46.2	2/ 7.7	–	–
The equipment was working properly	15/ 57.7	8/ 30.8	3/ 11.5	–	–
Tutor					
Tutor’s overall skills	22/ 84.6	2/ 7.7	1/ 3.8	1/ 3.8	-
Tutor’s professional performance	20/ 76.9	4/ 15.4	1/ 3.8	1/ 3.8	-
Tutor’s expertise	17/ 65.4	7/ 26.9	1/ 3.8	–	1/ 3.8
Tutor’s clarity	15/ 57.7	10/ 38.5	–	1/ 3.8	–
Tutor’s preparation of the sessions	24/ 92.3	1/ 3.8	–	1/ 3.8	–
Tutor’s time management	15/ 57.7	7/ 26.9	3/ 11.5	–	1/ 3.8
Tutor’s responsiveness	18/ 69.2	7/ 26.9	–	–	1/ 3.8

**Table 6:** The participants’ rating of the usefulness of the various aspects of the training (*N* = 26)

Statements	Very useful	Useful	Average	Useless	Very useless
	1	2	3	4	5
	Frequency (*n*)/ percentage (%)				
How useful did you find the pre-training quiz?	14/ 53.8	9/ 34.6	3/ 11.5	–	–
Did the pre-training quiz help you realise what you needed to learn during the training?	12/ 46.2	14/ 53.8	–	–	–
I found the training material pack that I received	17/ 65.4	8/ 30.8	1/ 3.8	–	–

**Table 7:** The participants’ agreement and satisfaction with various aspects of the training (*N* = 26)

Statements	Strongly agree	Agree	Neither agree or disagree	Disagree	Strongly disagree
	1	2	3	4	5
	Frequency (*n*)/ percentage (%)				
The purpose of the training was clear	13/ 50	13/ 50	–	–	–
The training content met my expectations	12/ 46.2	11/ 42.3	3/ 11.5	–	–
The topics presented in the training sessions was new to me	6/ 23.1	10/ 38.5	7/ 26.9	3/ 11.5	–
The training contributed to my understanding of obesity, obesity-related problems	8/ 30.8	16/ 61.5	2/ 7.7	–	–
The training improved my awareness about tools and resources available to detect and manage obesity	10/38.5	13/ 50	2/ 7.7	1/ 3.8	–
I fully understand the procedure of the weight management service that I will deliver	8/ 30.8	14/ 53.8	2/ 7.7	2/ 7.7	–
The training helped me understand my responsibilities in the weight management service I will deliver	9/ 34.6	15/ 57.7	–	2/ 7.7	–
I enjoyed the training	11/ 42.3	12/ 46.2	3/ 11.5	–	–

Statements	Very satisfied	Satisfied	Neutral	Dissatisfied	Very dissatisfied
	1	2	3	4	5
	Frequency (*n*)/ Percentage (%)				
Please rate your satisfaction level with the training organisation	15/ 57.7	9/ 34.6	2/ 7.7	–	–

Furthermore, pharmacists rated how useful the pre-training quiz was and how good the training materials were. They stated that the pre-training quiz was very useful/useful (88.4%, *n* = 23) and all pharmacists (100%, *n* = 26) noted that the pre-training quiz had helped them to realise their own educational needs. In addition, almost all pharmacists reported that the training materials received were very useful/useful (96.2%, *n* = 25) (Table 6).

All pharmacists (100%, *n* = 26) strongly agreed/agreed, citing that the purpose of the training was clear. They also added that their expectations were met, as 88.5% (*n* = 23) of them strongly agreed/agreed with this statement (Table 7).

During the training, participants also learned all the steps, procedures and responsibilities that they had to follow during the service delivery. Overall, 88.5% (*n* = 23) strongly agreed/agreed that they enjoyed the training indicating that they were very satisfied/satisfied with the training received 92.3% (*n* = 24) (Table 7).

#### Pharmacists’ perceived knowledge level pre- and post-training

The reliability for the knowledge level Likert-scale question (with 12 statements) was tested using Cronbach’s alpha resulting in 0.770 and 0.941, respectively (pre- and post-training), displaying good internal consistency. Table 8 illustrates how pharmacists rated their knowledge level before and after the training, including frequencies, the percentages of responses and the average for each statement requested.

**Table 8:** Pharmacists’ rating of their perceived knowledge level pre- and post-training

Before training						Statements	After training						
1	2	3	4	5	A*		1	2	3	4	5	A*A	A* difference
Excellent	Good	Average	Below average	Poor			Excellent	Good	Average	Below average	Poor		
Frequency (*n*) (1st row)/percentage (%) (2nd row)							Frequency (*n*) (1st row)/percentage (%) (2nd row)						
3	5	15	3	–	2.69	Scale of obesity	14	10	2	–	–	1.54	*1.15*
11.5%	19.2%	57.7%	11.5%				53.8%	38.5%	7.7%				
2	13	10	1	–	2.38	Consequences of obesity	17	8	1	–	–	1.38	*1*
7.7%	50%	38.5%	3.8%				65.4%	30.8%	3.8%				
1	5	14	6	–	2.96	Recommended daily calories intake	13	11	2	–	–	1.58	1.38
3.8%	19.2%	53.8%	23.1%				50%	42.3%	7.7%				
–	5	15	5	1	3.08	Recommended daily intake of foods per food groups	14	10	2	–	–	1.54	1.54
	19.2%	57.7%	19.2%	3.8%			53.8%	38.5%	7.7%				
–	11	11	4	–	2.73	Dietary recommendations	14	10	2	–	–	1.54	1.19
	42.3%	42.3%	15.4%				53.8%	38.5%	7.7%				
–	3	16	5	2	3.23	Alcohol consumption limits	10	12	4	–	–	1.77	1.46
	11.5%	61.5%	19.2%	7.7%			38.5%	46.2%	15.4%				
8	3	10	4	1	2.50	Calculation of BMI	16	9	1	–	–	1.42	*1.08*
30.8%	11.5%	38.5%	15.4%	3.8%			61.5%	34.6%	3.8%				
3	7	11	4	1	2.73	Measurement of waist circumference	15	9	2	–	–	1.50	1.23
11.5%	26.9	42.3	15.4	3.8			57.7%	34.6%	7.7%				
–	1	4	8	13	4.27	How to use AUDIT-C score	10	13	1	1	1	1.85	2.42
	3.8%	15.4%	30.8%	50%			38.5%	50%	3.8%	3.8%	3.8%		
–	11	13	1	1	2.69	Physical activity guidelines	14	11	1	–	–	1.50	1.19
	42.3%	50%	3.8%	3.8%			53.8%	42.3%	3.8%				
–	2	12	11	1	3.42	Motivational interviews and consultation skills	9	14	2	1	–	1.81	1.61
	7.7%	46.2%	42.3%	3.8%			34.6%	53.8%	7.7%	3.8%			
–	4	11	9	2	3.35	Weight management Programme overall	11	13	1	1	–	1.69	1.66
	15.4%	42.3%	34.6%	7.7%			42.3%	50%	3.8%	3.8%			
Overall A pre-training					3.003	Overall A post-training						1.593	

*A = average

Italics highlighting indicates the lowest average difference while underline indicates the maximum

Further analysis using the sign test showed that the training had a statistically significant effect on the perceived knowledge level (*P*-value < 0.001). Pharmacists’ total rating scores pre- and post-training are illustrated in Table 9. Interestingly, there was a statistically significant increase in the perceived knowledge level of pharmacists after the training (mean = 52.88, SD = 6.41), compared with the same level before the training (mean = 35.96, SD = 5.36) (*P*-value < 0.001).

**Table 9:** Pharmacists pre- and post-total scores of their perceived knowledge level

Q7 total scores		
Pharmacist code	Total score pre-training	Total score post-training
W1	32	53
W2	*25*	51
W3	34	54
W4	40	51
W5	37	55
W6	36	51
W7	43	60
W8	29	60
W9	34	48
W10	34	60
W11	40	60
W12	37	53
W13	39	45
W14	39	55
W15	32	60
W16	29	*33*
W17	28	59
W18	40	46
W19	44	51
W20	39	60
W21	35	45
W22	44	60
W23	41	53
W24	31	52
W25	43	51
W26	30	49
	Mean total score Pre-training	Mean total score Post-training
Means	35.96	52.88

The underline highlights the maximum total score observed before and after the training and the italics indicates the lowest total score

#### Pharmacists’ confidence level pre- and post-training

Upon checking the reliability of the confidence level scale pre- and post-training, the Cronbach’s alpha was 0.734 and 0.837 for pre- and post-training, respectively. From the results, there have been positive changes in pharmacists’ perceived confidence post training. Table 10 indicates the frequencies, percentages and means of pharmacists’ rating in relation to their confidence level before and after the training.

**Table 10:** Pharmacists’ rating of their perceived confidence level pre- and post-training

Before training						Statements	After training						
1	2	3	4	5	A*		1	2	3	4	5	A*	A* difference
Excellent	Good	Average	Below average	Poor			Excellent	Good	Average	Below average	Poor		
Frequency (*n*) (1st row)/percentage (%) (2nd row)							Frequency (*n*) (1st row)/percentage (%) (2nd row)						
2	10	12	2	–	2.54	I have confidence in approaching people	8	14	3	1	–	1.88	*0.66*
7.7%	38.5%	46.2%	7.7%				30.8%	53.8%	11.5%	3.8%			
1	11	12	2	–	2.58	I am confident to assess people’s weight	9	16	1	–	–	1.69	0.89
3.8%	42.3%	46.2%	7.7%				34.6%	61.5%	3.8%				
3	8	10	4	1	2.69	I am confident to measure people’s waist circumference	14	11	1	–	–	1.50	1.19
11.5%	30.8%	38.5%	15.4%	3.8%			53.8	42.3%	3.8%				
15	8	3	–	–	1.54	I am confident to measure blood pressure and heart rate	19	7	–	–	–	1.27 (	*0.27*
57.7%	30.8%	11.5%					73.1%	26.9%					
4	10	6	4	2	2.62	I have confidence and I am able to motivate people	10	11	3	2	–	1.88 (	*0.74*
15.4%	38.5%	23.1%	15.4%	7.7%			38.5%	42.3%	11.5%	7.7%			
1	7	13	3	2	2.92	I am confident to discuss and set personalised goals with them	6	13	5	1	1	2.15 (	0.77
3.8%	26.9%	50%	11.5%	7.7%			23.1%	50%	19.2%	3.8%	3.8%		
2	9	13	2	–	2.58	I am confident to follow them and to provide lifestyle advice	15	9	1	1	–	1.54	1.04
7.7%	34.6%	50%	7.7%				57.7%	34.6%	3.8%	3.8%			
Overall A pre-training					2.496	Overall A post-training						1.701	

*A = average

Italics highlighting indicates the lowest average difference while underline indicates the maximum

It is important to note that even if most pharmacists were already confident in taking BP and HR measurements prior to the training, there was a statistically significant increase in their perceived confidence level after the training. Furthermore, Table 11 indicates the minimum and the maximum total score pre- and post-training. There was a statistically significant increase in pharmacists’ perceived confidence level after their training (mean = 30.08, SD = 3.65) compared with before their training (mean = 24.54, SD = 3.82) (*P*-value < 0.001).

**Table 11:** Pharmacists’ total score pre- and post the training on their perceived confidence level

Q5 total scores		
Pharmacist code	Total score pre-training	Total score post-training
TW1	21	27
TW2	26	35
TW3	22	30
TW4	30	29
TW5	25	27
TW6	27	30
TW7	27	35
TW8	19	25
TW9	*17*	30
TW10	19	30
TW11	28	33
TW12	26	32
TW13	24	25
TW14	25	34
TW15	21	28
TW16	21	*21*
TW17	26	35
TW18	28	27
TW19	25	32
TW20	32	35
TW21	20	26
TW22	30	34
TW23	28	31
TW24	26	32
TW25	23	30
TW26	22	29
	Mean total score Pre-training	Mean total score Post-training
Means	24.54	30.08

The underline highlights the maximum total score observed before and after the training and the italics indicates the lowest total scores

### Pre- and post-training quiz

It was clear that pharmacists’ knowledge had improved after the training. It is important to note that pharmacists did not have access to the training materials when they completed the quiz. The quiz’s total score for each of the participating pharmacists had significantly improved after the training (*P*-value < 0.001). The mean total score of pharmacists had also increased (Table 12).

**Table 12:** The total scores achieved in the quiz pre- and post-training

Pharmacist code	Total score pre-training	Total score post-training*
TWM 1	3	13
TWM 2	4	11
TWM 3	8	12
TWM 4	5	12
TWM 5	7	*9*
TWM 6	7	11
TWM 7	10	12
TWM 8	11	14
TWM 9	8	11
TWM 10	4	12
TWM 11	7	13
TWM 12	7	12
TWM 13	*1*	12
TWM 14	7	13
TWM 15	4	13
TWM 16	7	13
TWM 17	11	12
TWM 18	8	13
TWM 19	6	10
TWM 20	3	12
TWM 21	4	13
TWM 22	8	10
TWM 23	7	13
TWM 24	9	10
TWM 25	7	13
TWM 26	3	12
	Mean total score Pre-training	Mean total score Post-training
Means	6.38 (SD = 2.56)	11.92 (SD = 1.20)

The underline highlights the maximum total score observed before and after the training and the italics indicates the lowest total score

A full analysis of the quiz responses is provided in Table 13 that shows the frequency with which participants responded correctly or incorrectly pre- and post-training and it also shows whether the training had a statistically significant outcome to each of the questions.

**Table 13:** Indicates the number of pharmacists who gave correct and wrong answer in the quiz and training’s effect (n/%)

Questions*			Correct (*n*/%)	Wrong (*n*/%)	*P* value	Training’s effect
Q1	How many adults in Greece were classified as obese according to World Health Organization (WHO)?	Pre—test	9/34.6	17/65.4	< 0.001	Positive
		Post-test	26/100	0		
Q2	What causes obesity?	Pre—test	23/ 88.5	3/11.5	0.25	No effect
		Post—test	26/100	0		
Q3	How much is the Body Mass Index (BMI) range that indicates that a person is obese?	Pre—test	13/50	13/50	< 0.001	Positive
		Post—test	26/100	0		
Q4	What is the recommended daily fruit and vegetable intake for an average adult according to the European Society of Cardiology (ESC) guidelines?	Pre—test	12/46.2	14/53.8	0.016	Positive
		Post—test	26/100	0		
Q5	(a) What is the recommended daily calorie intake for an average adult female?	Pre—test	7/26.9	19/73.1	0.004	Positive
		Post—test	20/76.9	6/23.1		
	(b) What is the recommended daily calorie intake for an average adult male?	Pre—test	8/ 30.8	18/69.2	0.004	Positive
		Post—test	21/ 80.8	5/19.2		
Q6	What is the acceptable daily intake of saturated fats?	Pre—test	19/73.1	7/29.9	< 0.001	Positive
		Post—test	26/100	0		
Q7	Some fats have been shown to increase cardiovascular disease risk. Which one(s)?	Pre—test	6/23.1	20/ 76.9	< 0.001	Positive
		Post—test	25/96.2	1/3.8		
Q8	An obese person has excess weight that includes muscle, bone fat and water	Pre—test	16/ 61.5	10/ 38.5	0.18	No effect
		Post—test	22/84.6	4/15.4		
Q9	What is the alcohol daily limit for an adult male according to the European Society of Cardiology (ESC) guidelines?	Pre—test	10/38.5	16/61.5	< 0.001	Positive
		Post—test	24/ 92.3	2/7.7		
Q10	Alcohol contains more calories per unit weight than carbohydrates	Pre—test	12/46.2	14/53.8	1	No effect
		Post—test	13/50	13/50		
Q11	How many calories does a medium glass of red wine (175 ml) have?	Pre—test	11/42.3	15/ 57.7	< 0.001	Negative
		Post—test	3/11.5	23/88.5		
Q12	How much is the recommended weekly physical activity for an average adult, according to the European Society of Cardiology (ESC) guidelines?	Pre—test	13/50	13/50	0.021	Positive
		Post-test	26/100	0		
Q13	Motivational interviewing deals with which of the following four points?	Pre—test	7/26.9	19/73.1	< 0.001	Positive
		Post—test	26/100	0		

*McNemar’s test^+^ was conducted. All questions were statistically significant (*P* value < 0.05) apart from Q2, Q8 and 10

^+^For the test, the answers were treated either as correct or incorrect (an unanswered question was treated as an incorrect answer)

## Discussion

To our knowledge, this was the first and only study conducted to design a training model and evaluate a formal postgraduate training programme for Greek pharmacists, allowing them to expand their clinical role in offering pharmacy-led public health services.

Pharmacists’ current knowledge was assessed through the pre-training quiz given. This was conducted in the same way as the Bajorek et al. [38] study, which trained pharmacists to deliver a service for hypertensive patients, showing that it is important to carefully examine the pre-existing knowledge of pharmacists before they are given any further education. This is because it is sometimes assumed that pharmacists have the same educational background when this is not always the case. Without further examination, this may have certain implications for the training. Additionally, it was important that the perceived and actual knowledge levels were assessed as according to Stoutenborough et al. [39] both should be used as an indication of knowledge as they do not capture scientific knowledge in the same way.

The training itself was based on the pharmacists’ training needs including lectures and active learning techniques. The duration of the lectures was kept to a minimum for two reasons: first because time constraints are a major concern of pharmacists, and secondly because we wanted to give participants the opportunity to discuss obesity and WM with the tutor (researcher) and their peers. Simulated learning (such as role-playing), included in experiential learning, was used, since it is a tool that facilitates professionals’ interaction [40]. Additionally, role-playing was used to inspire pharmacists to learn problem-solving in a patient-centred approach, to help them improve their communication skills through discussion, and to help them raise their self-confidence through the new information they received during the training [23]. It also enabled pharmacists to prepare themselves in a safe environment to practise, so they would be ready for delivery the actual WM programme.

Pharmacists were positive about the training, claiming it met their expectations and almost all of them stated that they had gained a generally positive experience from the training. All in all, the training successfully increased pharmacists’ perceived confidence and knowledge levels on obesity and WM. The results of the evaluation questionnaire showed that the training significantly increased their perceived confidence; not only in approaching people (*P*-value < 0.001), but also when taking people’s anthropometric measurements; BP and HR (*P*-value = 0.031). Furthermore, pharmacists were also eager to set personalised goals with their clients so that they could motivate them to make a behaviour change, but also so that they could offer them advice to improve their well-being. The increased perceived confidence levels observed by the participating pharmacists were echoed by other studies [20, 41–43]. All studies noted that further education increases the confidence of HCPs when offering their clients advice on health issues. Furthermore, because of the training there was a statistically significant improvement in perceived knowledge levels (*P*-value < 0.001).

In addition, the same quiz was given to pharmacists before and after the training to determine whether there were any changes in their knowledge levels, as well as to determine whether the training had a positive effect overall. Interestingly, what pharmacists shared during the interviews regarding their educational needs reflected their given responses in the pre-training quiz, showing that they were aware of their weaknesses. The questions that had the lowest scores were related to calorie-intake recommendations, motivational interviewing and type of fats associated with CVD risk. The post-training quiz confirmed that a significant increase in knowledge level (*P*-value < 0.001) was observed as a result of the training. Before the training the mean total score in the quiz was 6.38, whereas after the training it was 11.92. However, a possible reason for this could be that participating pharmacists’ knowledge was still fresh as the quiz was completed by each one of them at the end of the training.

The findings of Sinclair et al. [12] suggest that consistent training should be provided to pharmacists on health promotion issues, something that turned out to be different judging by the comments received from Greek pharmacists. Although an adequate number of pharmacists (*n* = 11) agreed that repeating the training would be beneficial, there were pharmacists who stated that they did not need to repeat the training. A possible reason for this may be the fact that the training was still ‘fresh’, and they may have felt that they had covered everything during the training.

The study had several limitations. First, it only explored the short-term effects of the training on pharmacists since the evaluation questionnaire was given straight after the second session. The long-term effects were not examined, as knowledge might decrease after a period. This accords with the Gass et al. study [44], which examined the knowledge retention and skill of physicians and nurses after training in cardiopulmonary resuscitation. The study’s findings revealed that there was a significant reduction in knowledge and skill 6 months after training and that there was also a further reduction in basic life support skills after 12 months of the training. Additionally, the training’s evaluation was conducted by the organiser (researcher) which could have biased the findings. Sinclair et al. [12] states that evaluation should ideally be performed by an “objective outsider”. Another limitation was that a small sample size was used, due to the number of pharmacists who agreed to participate in the programme, as well as to the location of the study. While Patras is the third largest city in Greece, more pharmacists might have been interested in participating if it had taken place in a larger city, such as Athens. Furthermore, the fact that the same quiz was given to the pharmacists before and after the training, could act as a limitation, as pharmacists have seen the questions before and might have researched the answers, thus improving their results to the quiz post training.

## Conclusions

Generally, the results indicate that the training was effective and had positive outcomes. In collaboration with the Greek-speaking Pharmacy Courses, the Panhellenic Pharmaceutical Association should collaborate and offer continuous training to pharmacists to enable them to provide such services. In general, due to the positive outcomes of the training, however, it has the potential to become a model for Greek pharmacists to offer different pharmacy-led public health services in Greece.

## Data Availability

The authors are happy to share any of the raw materials to pharmacists wishing to use them.
